# Temporal Trends in Incidence of Nutritional Deficiency among Older Adults in China: A Secondary Analysis of the Global Burden of Disease Study 1990–2019

**DOI:** 10.3390/nu14235008

**Published:** 2022-11-25

**Authors:** Linqi Xiao, Jialin Fu, Likai Lin, Yong Han

**Affiliations:** 1 Hospital Management Institute of Wuhan University, Zhongnan Hospital of Wuhan University, Wuhan 430071, China; 2 Department of Preventive Medicine, School of Health Sciences, Wuhan University, Wuhan 430071, China

**Keywords:** nutritional deficiency, temporal trends, incidence, older adults, Chinese

## Abstract

Nutritional deficiency is prevalent among the elderly, and it is associated with many adverse health consequences. China is rapidly moving toward an aging society with a large population; however, evidence on the epidemiological trends in nutritional deficiency among the Chinese elderly is limited. Data on the incidence of nutritional deficiency among Chinese adults aged 65 years or above from 1990 to 2019 were extracted from the *Global Burden of Disease 2019* database. We used the joinpoint regression method to estimate the average annual percentage change (AAPC) and to describe trend patterns. Age, period, and cohort effects were determined using age–period–cohort models. From 1990 to 2019, the incidence of vitamin A deficiency and iodine deficiency among Chinese older adults decreased from 1784.12 and 8.20 to 304.27 and 7.26 per 100,000, with AAPCs of −0.41 (−0.44, −0.38)% and −5.86 (−6.29, −5.43)%, respectively. A continually increasing trend was seen for incidence rates of protein-energy malnutrition, from 1342.02 to 2275.87 per 100,000 person-years, with an AAPC of 1.70 (1.40, 2.01)%. These trends were more pronounced among men than women. A strong age effect and birth cohort effect were present. Specifically, the population that was older or born later had a lower incidence of deficiencies in vitamin A and iodine but a higher incidence of protein-energy malnutrition. The results show a substantial reduction in vitamin A and iodine deficiencies among the Chinese elderly, and health policies and public awareness are needed to address the burden of protein-energy malnutrition in this population.

## 1. Introduction

The Chinese population is aging rapidly. According to the results of the seventh Chinese population census, there were 264 million people aged 60 and over in 2020, accounting for 18.7% of the total population of China [1]. The number is expected to reach 402 million (or 28% of the total population) by 2040, as estimated by the World Health Organization [2]. Aging is the primary driver of most chronic diseases and declined quality of life [3]. It is crucial to address and prepare for the challenges and issues accompanied by the aging society in China.

Nutritional deficiency has been known to be one of the main influences of healthy aging. Although total energy intake declines with age, the requirements for many nutrients increase to maintain organ systems with declining functionality [4]. The elderly tend to have more trouble with digestion and with the absorption of food and have a lower intake of nutrient-rich foods due to their oral health, inability to chew, mouth dryness, and decreased appetite, increasing their risk of nutritional deficiency [5]. It is therefore more difficult for the elderly to meet their nutrient requirements than it is for younger adults. Nutritional deficiency in the elderly has a major impact on disease and disability, including anemia, frailty, blindness, osteoporosis, cardiovascular disease, and diabetes [6]. During the past three decades, from 1990 to 2019, there was an overall decreasing trend in the prevalence of vitamin A deficiency and iodine deficiency but an increasing trend in the prevalence of protein-energy malnutrition worldwide although great differences existed across different regions and populations [7,8,9]. With rapid economic growth and urbanization, Chinese dietary patterns have changed considerably from a predominantly plant-based diet to one that includes large amounts of animal products, a lower intake of cereals and vegetables, and higher consumption of red meat, processed meat, and sugar-sweetened beverages. These dietary changes, combined with the rapid growth of the aging population, suggest a potentially temporal change in the nutritional status among the elderly in China.

Previous studies on the epidemiological characteristics of nutritional deficiency among the elderly in China have primarily been conducted in hospital settings [10] or in a specific region of China [11]. Based on the Global Burden of Disease (GBD) study conducted in 1990–2019, Leung et al. depicted the temporal trends in disability-adjusted life years and years of healthy life lost due to disability from overall nutritional deficiency among the elderly in the Western Pacific region [12]. Chen et al. examined the changes in the prevalence of deficiency in specific micronutrients, such as vitamin A, iodine, and dietary iron, among all age groups in China [13]. However, evidence on the incidence of nutritional deficiency among the elderly in China remains scarce, which is crucial to guide future research, policy making, and prevention strategies.

In this study, we aimed to investigate the temporal trends in the incidence of common nutritional deficiencies among the elderly in China over the past three decades using data from the GBD study carried out in 1990–2019.

## 2. Materials and Methods

### 2.1. Data Sources and Definitions

To understand what the most important contributors to health loss in a given place, time, and age–sex group are, the World Health Organization and the World Bank developed the GBD research program using a set of continually improving estimation methods and all available epidemiological data to produce comprehensive, internally consistent, and comparable sets of estimates of mortality and disability from disease and injury for all regions of the world [14]. The GBD 2019 provides full time-series estimates of the incidence, prevalence, mortality, years lived with disability, years of life lost, and disability-adjusted life years from 1990 to the present for 369 diseases and injuries, men and women, various age groups, 21 regions, and 204 countries and territories from 1990 to 2019 [15]. Details on the methodology used in the GBD study can be found elsewhere [15,16].

Nutritional deficiencies in the GBD 2019 included vitamin A deficiency, iodine deficiency, dietary iron deficiency, protein-energy malnutrition, and other nutritional deficiencies [15]. Vitamin A deficiency was defined as a serum retinol <0.7 μmol/L and/or the presence of blindness and vision loss due to vitamin A deficiency. Iodine deficiency included estimates of the subset of iodine deficiency associated with visible goiter (grade 2) and its associated sequelae, including thyroid dysfunction, heart failure, and intellectual disability (historically referred to as “cretinism”). As the estimates of incidence for dietary iron deficiency among people in China were 0, we did not include dietary iron deficiency in this study. Protein-energy malnutrition included moderate and severe acute malnutrition. It was defined in terms of weight-for-height Z-scores according to the WHO 2006 growth standard. The GBD 2019 used the International Classification of Diseases codes (ICD 10 codes E40–E46.9 and E64.0, and ICD 9 codes 260–263.9), the official system of assigning codes to diagnoses and procedures associated with hospital utilization in the United States [17], to identify protein-energy malnutrition. Other nutritional deficiencies encompassed a wide variety of causes of morbidity, ranging from vitamin deficiencies to other nutritional anemias. The GBD 2019 extracted data from the WHO Vitamin and Mineral Nutrition Information System. It also performed a systematic review of the data published in *PubMed*, searched the Global Health Data Exchange for multi-country and national surveys, and contacted other research groups to capture any remaining data sources. For studies reporting incidence for both sexes combined, estimates were split by sex using the within-study sex ratio or the sex ratio from a meta-analysis of existing sex-specific data conducted by the GBD 2019 research group [15]. Reported estimates for broad age groups were split into five-year age groups using the age pattern estimated by DisMod-MR 2.1 developed by the GBD 2019 [15].

In this study, we used the GBD results tool to obtain annual age-standardized and sex-specific estimates of incidence with 95% uncertainty intervals from 1990 to 2019 (2019 is the last date, updated in 2022) [18] for parent nutritional deficiency and specific deficiencies, including vitamin A deficiency, iodine deficiency, and protein-energy malnutrition, among Chinese individuals aged 65 or above.

### 2.2. Statistical Analysis

The chi-square test was used to compare incidence rates between sex groups. Joinpoint regression analysis was used to describe the age-specific and sex-combined temporal trends in the incidence of nutritional deficiencies and to detect the time points at which significant changes occurred. A maximum of five joinpoints was allowed in the models based on the number of observations (30 calendar years), and a Monte Carlo permutation method was used for model selection [19]. We set the minimum number of observations from a joinpoint to either end of the data or between two joinpoints as three to reduce over-fitting of the models. We used the Durbin–Watson test to examine the autocorrelation. A sensitivity analysis correcting the autocorrelation parameters in joinpoint regression models was also conducted to evaluate potential impact of autocorrelation. The annual percentage change (APC) for each linear trend segment detected from the joinpoint regression model and the average annual percentage change (AAPC) for the entire study period were estimated. A pairwise comparison of AAPC between sex groups was conducted using the joinpoint regression model.

The age–period–cohort model was developed to examine the effects of age, period, and birth cohort on the incidence rates of nutritional deficiencies. In the age–period–cohort model, individuals were divided into consecutive 5-year age groups (65–69, 70–74, …, 80–84, 85–89), consecutive 5-year periods (1994, 1999, …, 2014, and 2019), and corresponding consecutive 5-year birth cohort groups (1905–1909, 1910–1914, …, 1945–1949, and 1950–1954). The effects of age, period, and birth cohort were measured by the exponential value of the estimated coefficients, which refers to the age-specific incidence rates for age effect and relative risk (RR) for the period effect and birth cohort effect.

Statistical significance was set at a *p*-value of <0.05. All analyses were conducted using R software, version 4.1.2 (R Foundation for Statistical Computing, Vienna, Austria) or Joinpoint Regression Program, version 4.9.0.0 (Statistical Methodology and Applications Branch, Surveillance Research Program, National Cancer Institute, Bethesda, MD, USA).

## 3. Results

### 3.1. Temporal Trends in Nutritional Deficiencies

**Figure 1:**
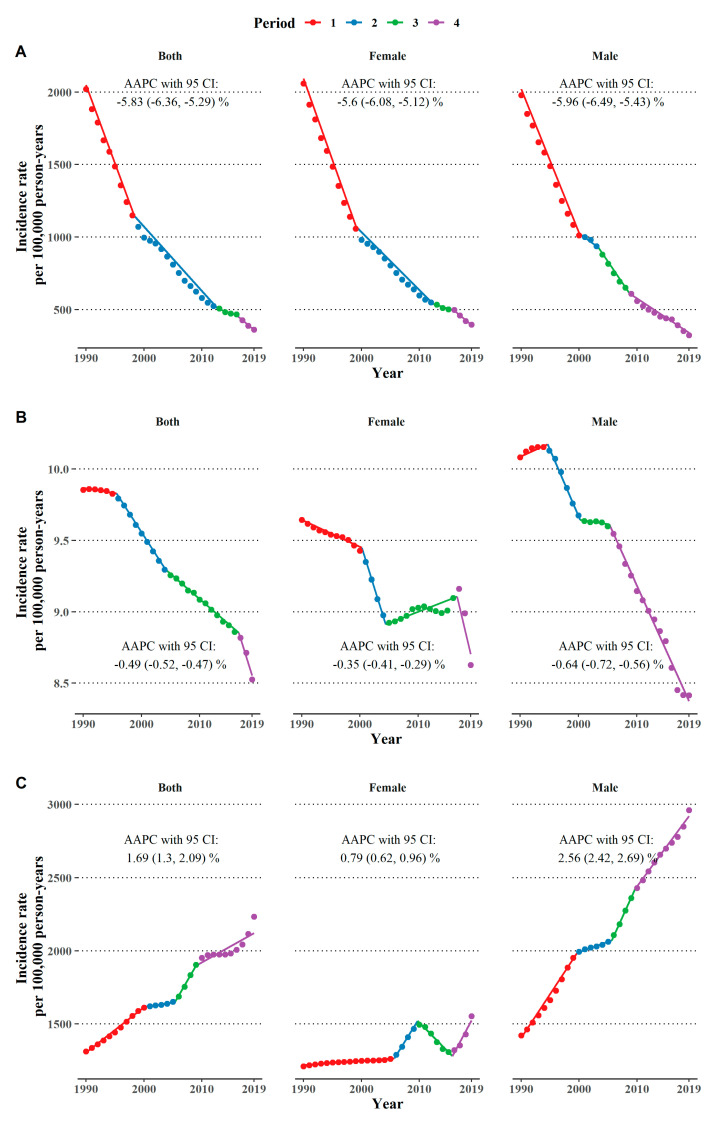
Trends in age-standardized incidence rate per 100,000 person-years of (**A**) vitamin A deficiency, (**B**) iodine deficiency, and (**C**) protein-energy malnutrition among the elderly in China from 1990 to 2019, as stratified by sex.

The past three decades have seen a decline in the age-standardized incidence of vitamin A deficiency among the elderly in China, from 2020.39 per 100,000 person-years in 1990 to 360.43 per 100,000 person-years in 2019. The AAPC with a 95% CI was −5.83 (−6.32, −5.29)% (Figure 1A). A moderate decreasing trend has been observed since 2000 in both males and females, as shown in Figure 1A and Table S1. Compared to women, men had lower incidence rates (for instance, 1978.78 vs. 2058.56 per 100,000 person years in 1990, *p*-value < 0.001) and a larger AAPC (−5.96% vs. −5.60%) although statistical significance was not reached (*p*-value = 0.330).

In 1990, the age-standardized incidence of iodine deficiency among the Chinese elderly was 9.85 per 100,000 person-years; the number decreased to 8.53 per 100,000 person years in 2019, with an APPC of −0.49 (−0.52, −0.47)% (Figure 1B). Men had a higher incidence rate in 1990 (10.08 vs. 9.64 per 100,000 person-years, *p*-value < 0.001), a significantly larger AAPC (−0.64% vs. −0.35%, *p*-value < 0.001), and eventually, a lower incidence rate (8.42 vs. 8.63 per 100,000 person years, *p*-value < 0.001) compared to women in 2019. During the first decade, the incidence rate reached its peak in 1994 and then dropped among men, while the incidence continually decreased among women. Men have maintained a declining trend in the last two decades, while women showed an increasing trend from 2004 to 2017, with an APC of 0.18 (0.12, 0.23)% (Figure 1B and Table S1).

From 1990 to 2019, the age-standardized incidence rate of protein-energy malnutrition increased from 1309.37 per 100,000 person-years to 2232.90 per 100,000 person-years, with an AAPC of 1.69 (1.30, 2.09)%. Men had higher incidence rates (for instance, 1419.38 vs. 1208.49 per 100,000 person-years, *p*-value < 0.001) and a faster speed of increase than women (AAPC: 2.56% vs. 0.79%, *p*-value < 0.001). A continually increasing trend was seen among men, except for in the period from 2000 to 2006, during which the trend became flat. As for women, the increase in the incidence rates accelerated after a 15-year period of sluggish growth and peaked around 2010; then, a U-shaped trend was seen during the last decade (Figure 1C and Table S1).

The results of the Durbin–Watson test are shown in Table S2. Autocorrelation existed in most of the models (*p*-value < 0.05) except for the analysis of iodine deficiency in both sexes combined and protein-energy malnutrition among females. After correcting for autocorrelation in the models, the number of joinpoints decreased for trends in vitamin A deficiency but remained for trends in other deficiencies (Figure S1).

### 3.2. The Effect of Age, Period, and Cohort

Table 1 depicts the effects of age, period, and cohort on the incidence rates. The age effect and the age-specific incidence rates per 100,000 person-years controlled for period effect and cohort effect showed a continuously decreasing trend along with the older age groups among both sex groups for vitamin A deficiency and iodine deficiency, but the reverse was observed for protein-energy malnutrition, which presented a positive association between age effect and incidence rates.

For vitamin A deficiency and protein-energy malnutrition, the period effects were statistically significant although the magnitude of the effect size was small, and no clear change trend was found. No significant period effect was observed for iodine deficiency.

Similar to the age effect, the relative risk of vitamin A deficiency and iodine deficiency decreased with the recency of birth cohort from 1905 to 1954; however, the opposite situation was observed for protein-energy malnutrition.

## 4. Discussion

Using data from the GBD, we provided a comprehensive picture of the temporal trends in the incidence rates of nutritional deficiencies among the elderly in China from 1990 to 2019 for the first time. Overall, we revealed substantial declines in the incidence rates of vitamin A deficiency and iodine deficiency, with the exception of protein-energy malnutrition, which presented a continuously increasing pattern. These changes were more marked among men than women. A strong age effect and birth cohort effect were observed in older people, and those who were born later had a lower incidence of deficiencies in vitamin A and iodine but a higher incidence of protein-energy malnutrition.

### 4.1. Trends in Vitamin A Deficiency

The continual decline in the incidence rates of vitamin A deficiency observed among the Chinese elderly is consistent with the trends presented in the younger Chinese population [20] and in their older counterparts in other developing regions [7]. The primary reason for the improvement in preventing vitamin A deficiency is the economic development of China. Han et al. demonstrated that the burden of vitamin A deficiency on a population is negatively associated with a higher socio-demographic index and the availability and quality of healthcare [20]. Since China began to open up and reform its economy in 1978, there have been significant improvements in access to healthcare and education [21,22], and there has been a transformation in dietary patterns. According to the China Health and Nutrition Survey, dietary patterns with high intakes of egg, dairy products, and meat have increased over the past three decades, especially among men [23], and these food sources represent the most important sources of retinol for Chinese adults [24]. Moreover, China has formulated a series of nutritional plans and actions aiming to eliminate nutritional deficiencies and to promote consumption of foods rich in carotene and retinol, which were included in the national economic and social development program [25].

### 4.2. Trends in Iodine Deficiency

We observed an overall decrease in the incidence rates of iodine deficiency among the Chinese elderly. The Chinese Iodine Deficiency Disorder Elimination Programme (CIDDEP) has played the most important role in this improvement. Taking mandatory universal salt iodization as the main strategy, the CIDDEP established a comprehensive framework for the prevention, management, surveillance, and evaluation of iodine deficiency [26]. At the time of the 2011 National Iodine Deficiency Disorder survey, 95.3% of households were consuming salt with 20–50 ppm of iodine (the Chinese national standard) [27]. However, we also noticed a rebound in the incidence rates of iodine deficiency among the female elderly in China from 2004 to 2017, which might be related to the adjustment of decreasing the concentration of iodized table salt in some provinces in recent years [28]. Moreover, due to changes health consciousness, there was a reduction in salt consumption, especially among women [29], which might have also resulted in a decline in iodine intake. The fluctuating trend among women during the past few decades indicates that government policies should pay more attention to iodine deficiency among the elderly population. As for the dramatic decline since 2017 among females, there is no reasonable explanation. However, it is worth noting that the national-level data sources of the GBD 2019 study for nutritional deficiency among elderly Chinese people were conducted up to 2017, and incidence rates in 2018 and 2019 were estimated via a modelling method based on datasets at the level of city, which may be susceptible to the bias of statistical parameters and geographic variation.

### 4.3. Trends in Protein-Energy Malnutrition

This study suggests an alarmingly increasing trend in the incidence of protein-energy malnutrition among the Chinese elderly, which is in line with previous studies in Chinese people and in other populations worldwide [8,30]. Although China’s economic development has greatly improved people’s ability to access food and nutrition knowledge, older adults consumed significantly less daily protein from 1991 to 2018, especially among men when compared to women (with an annual change of −0.034 g versus −0.030 g) [31]. The increasing trend in the incidence of protein-energy malnutrition might also be explained by the continually increasing life expectancy in China from 61 years in 1990 to 78 years in 2019 [32]. With a longer expected lifetime, more people are at risk of protein-energy malnutrition when they age. Moreover, with increasing nutrition screening programs and more comprehensive measurement items included in health surveys [33], people with undiagnosed protein-energy malnutrition have a greater opportunity for this to be detected, which might also contribute to the rise in the incidence of protein-energy malnutrition among the elderly.

Our results observed a strong age effect in which older people had a higher incidence of protein-energy malnutrition. This is easy to understand given the physiological and psychosocial problems that emerge with aging. Firstly, decreased appetite among the elderly influences protein and total energy intake [34,35]. Secondly, dementia, dysphagia, and other physical dysfunctions that are prevalent in older adults also cause difficulties in eating and drinking [36]. Moreover, the nutrition knowledge and nutrition attitudes of the elderly in China still need to be improved [37]. Our study revealed a birth cohort effect that later birth cohorts had a higher incident risk of protein-energy malnutrition. Such a trend indicates the burden of protein-energy malnutrition in Chinese older adults has not been emphasized.

It is worth noting that the incidence rate of protein-energy malnutrition and that of vitamin A deficiency changed in opposite directions among elderly Chinese people although these two nutritional deficiencies are closely connected with one another. The cause of this seemingly contradictory result is unclear. One possible explanation is that the government in China attached more attention to micronutrient deficiency versus protein-energy malnutrition in the elderly. For instance, the National Health Commission only mentioned micronutrient deficiency regarding outstanding nutrition issues in the elderly at the press conference for the Report on Chinese Residents’ Chronic Diseases and Nutrition 2020 [38]. This reflects the fact that policies and programs on nutrition improvement in elderly Chinese people give higher priority to the prevention and treatment of micronutrient deficiency versus protein-energy malnutrition. However, caution is still required when interpretating this finding, considering the possible bias introduced by the data sources and statistical modelling in the GBD 2019.

This study calls for urgent and effective actions from the government and individuals to address the prevention, early identification, and management of protein-energy malnutrition among the elderly in China. Specifically, health policymakers should call attention to the harms and burden of protein-energy malnutrition in older adults and promote the health literacy of older people via video content, press releases, or social media. It is also recommended that the government implement routine screenings for malnutrition, improve dental health in the elderly, subsidize the price of food for the elderly, and increase the ability to access high-protein food. It is necessary to provide nutrition support for malnourished people who are unable to maintain their body weight by food intake alone. Elderly people with protein-energy malnutrition and their caregivers are encouraged to improve their awareness of and basic knowledge about nutritional problems, thus promoting the adequate consumption of dairy products or shakes containing protein supplements [39].

### 4.4. Limitations

There are several limitations in this study. Firstly, the incidence rate for a given region at a certain period was estimated using a Bayesian hierarchy model based on available data obtained from an extensive systematic review [15]. The accuracy of the estimates largely depends on the statistical assumptions and the data sources [15]. Although the data from the GBD study claim to be of high quality, differences in data collection, extraction, and the coding of data sources inevitably introduce bias. Secondly, there is a lack of data on deficiencies in other important nutritional elements, such as vitamin C, calcium, and folate, and further studies are needed to assess their epidemiological burden and trends in Chinese elderly. Thirdly, data are only available at the national level in the GBD study, whereas China has a socio-economically and topographically diverse landscape, and dietary patterns are also unevenly distributed. Further studies are needed to examine the geographical variations in the incidence rates of nutritional deficiencies among the Chinese elderly.

## 5. Conclusions

This study provides an insight into the trends in the incidence of nutritional deficiencies among elderly Chinese individuals. From 1990 to 2019, elderly Chinese people experienced a substantial reduction in incident cases of vitamin A deficiency and iodine deficiency due to economic development and the implement of nutritional intervention programs in China. However, the burden of protein-energy malnutrition in older Chinese adults should attract more attention. Urgent and effective actions from the government and individuals, such as enhancing the awareness of and education regarding protein-energy malnutrition, improving health literacy and nutrition, implementing routine screenings for malnutrition, increasing the ability to access high-protein food, and early treatment, are required to address the prevention, early identification, and management of protein-energy malnutrition among the elderly in China.

## Figures and Tables

**Table 1:** The effect of age, period, and cohort on incidence of vitamin A deficiency, iodine deficiency, and protein-energy malnutrition among the elderly in China from 1990 to 2019, as stratified by sex.

		Both Sexes Combined	Male	Female
Vitamin A deficiency	Age effect: Age-specific incidence rate per 100,000 person-years (95% CI)			
	65–69	8349.81 (8256.74, 8443.93)	8632.09 (8473.00, 8794.17)	8148.16 (8034.02, 8263.93)
	70–74	5007.98 (4953.66, 5062.89)	5143.90 (5051.42, 5238.08)	5005.56 (4937.32, 5074.73)
	75–79	3244.68 (3211.96, 3277.74)	3164.02 (3111.58, 3217.35)	3330.35 (3288.16, 3373.09)
	80–84	2213.25 (2193.00, 2233.70)	1961.68 (1931.61, 1992.22)	2336.82 (2310.09, 2363.85)
	85–89	1522.70 (1509.39, 1536.13)	1216.24 (1198.29, 1234.45)	1654.28 (1636.34, 1672.41)
	Period effect: RR (95% CI)			
	1994	1.06 (1.05, 1.06)	1.01 (1.00, 1.01)	1.09 (1.09, 1.09)
	1999	0.94 (0.94, 0.94)	0.95 (0.95, 0.95)	0.93 (0.93, 0.93)
	2004	1.02 (1.02, 1.02)	1.05 (1.05, 1.06)	0.99 (0.99, 1.00)
	2009	0.99 (0.99, 1.00)	1.01 (1.01, 1.01)	0.99 (0.98, 0.99)
	2014	0.98 (0.98, 0.98)	0.98 (0.98, 0.98)	0.99 (0.99, 0.99)
	2019	1.02 (1.01, 1.02)	1.00 (1.00, 1.00)	1.02 (1.02, 1.02)
	Cohort effect: RR (95% CI)			
	1905–1909	0.64 (0.64, 0.64)	0.64 (0.63, 0.64)	0.64 (0.64, 0.65)
	1910–1914	0.47 (0.46, 0.47)	0.46 (0.46, 0.47)	0.47 (0.47, 0.47)
	1915–1919	0.34 (0.34, 0.34)	0.33 (0.33, 0.34)	0.34 (0.34, 0.35)
	1920–1924	0.25 (0.24, 0.25)	0.24 (0.24, 0.25)	0.25 (0.25, 0.25)
	1925–1929	0.18 (0.18, 0.18)	0.18 (0.17, 0.18)	0.18 (0.18, 0.19)
	1930–1934	0.14 (0.13, 0.14)	0.13 (0.13, 0.13)	0.14 (0.14, 0.14)
	1935–1939	0.10 (0.10, 0.10)	0.09 (0.09, 0.10)	0.11 (0.11, 0.11)
	1940–1944	0.08 (0.08, 0.08)	0.07 (0.07, 0.07)	0.08 (0.08, 0.08)
	1945–1949	0.06 (0.06, 0.06)	0.05 (0.05, 0.05)	0.06 (0.06, 0.07)
	1950–1954	0.04 (0.04, 0.04)	0.04 (0.04, 0.04)	0.05 (0.05, 0.05)
Iodine deficiency	Age effect: Age-specific incidence rate per 100,000 person-years (95% CI)			
	65–69	11.40 (10.34, 12.56)	11.75 (10.10, 13.67)	10.91 (9.49, 12.55)
	70–74	8.04 (7.30, 8.85)	8.31 (7.15, 9.64)	7.60 (6.62, 8.72)
	75–79	6.35 (5.80, 6.96)	6.45 (5.60, 7.44)	5.98 (5.23, 6.82)
	80–84	5.17 (4.74, 5.64)	5.13 (4.47, 5.89)	5.10 (4.50, 5.78)
	85–89	4.20 (3.85, 4.59)	4.08 (3.55, 4.69)	4.41 (3.89, 5.00)
	Period effect: RR (95% CI)			
	1994	0.99 (0.97, 1.02)	0.99 (0.96, 1.03)	1.00 (0.97, 1.03)
	1999	1.00 (0.99, 1.01)	1.00 (0.98, 1.02)	1.00 (0.99, 1.02)
	2004	1.01 (0.99, 1.02)	1.01 (0.98, 1.03)	1.01 (0.98, 1.03)
	2009	1.01 (0.98, 1.03)	1.01 (0.98, 1.04)	1.00 (0.97, 1.03)
	2014	0.99 (0.97, 1.01)	0.99 (0.96, 1.01)	0.99 (0.97, 1.02)
	2019	1.00 (0.99, 1.02)	1.00 (0.98, 1.02)	1.01 (0.99, 1.02)
	Cohort effect: RR (95% CI)			
	1905–1909	0.98 (0.96, 1.01)	0.97 (0.94, 1.01)	0.99 (0.96, 1.03)
	1910–1914	0.97 (0.93, 1.01)	0.96 (0.90, 1.02)	0.99 (0.93, 1.05)
	1915–1919	0.95 (0.90, 1.01)	0.94 (0.86, 1.03)	0.98 (0.90, 1.08)
	1920–1924	0.94 (0.87, 1.02)	0.92 (0.82, 1.04)	0.98 (0.87, 1.10)
	1925–1929	0.93 (0.85, 1.02)	0.91 (0.78, 1.05)	0.97 (0.84, 1.12)
	1930–1934	0.91 (0.82, 1.01)	0.89 (0.76, 1.05)	0.95 (0.82, 1.10)
	1935–1939	0.88 (0.80, 0.98)	0.87 (0.74, 1.01)	0.93 (0.81, 1.06)
	1940–1944	0.88 (0.80, 0.97)	0.85 (0.73, 0.99)	0.94 (0.82, 1.08)
	1945–1949	0.86 (0.78, 0.95)	0.81 (0.70, 0.95)	0.92 (0.80, 1.06)
	1950–1954	0.81 (0.74, 0.90)	0.77 (0.66, 0.90)	0.87 (0.76, 1.00)
Protein-energy malnutrition	Age effect: Age-specific incidence rate per 100,000 person-years (95% CI)			
	65–69	865.03 (859.87, 870.22)	819.75 (813.69, 825.85)	932.61 (924.60, 940.68)
	70–74	977.33 (971.54, 983.16)	959.37 (952.33, 966.46)	1011.25 (1002.63, 1019.93)
	75–79	1103.14 (1096.77, 1109.55)	1127.64 (1119.54, 1135.80)	1097.40 (1088.35, 1106.53)
	80–84	1212.03 (1205.32, 1218.77)	1324.00 (1314.84, 1333.23)	1186.71 (1177.43, 1196.07)
	85–89	1322.22 (1314.76, 1329.71)	1553.67 (1542.48, 1564.93)	1282.03 (1271.89, 1292.25)
	Period effect: RR (95% CI)			
	1994	1.01 (1.01, 1.01)	1.01 (1.01, 1.01)	1.05 (1.04, 1.05)
	1999	1.00 (1.00, 1.00)	1.00 (1.00, 1.00)	0.98 (0.98, 0.98)
	2004	0.99 (0.99, 0.99)	0.99 (0.99, 0.99)	0.96 (0.96, 0.96)
	2009	0.99 (0.99, 0.99)	0.99 (0.99, 1.00)	1.06 (1.06, 1.06)
	2014	1.00 (1.00, 1.00)	1.01 (1.01, 1.01)	0.93 (0.93, 0.93)
	2019	1.01 (1.01, 1.01)	1.00 (1.00, 1.00)	1.03 (1.03, 1.04)
	Cohort effect: RR (95% CI)			
	1905–1909	1.13 (1.13, 1.13)	1.20 (1.20, 1.20)	1.06 (1.06, 1.06)
	1910–1914	1.24 (1.24, 1.24)	1.36 (1.36, 1.37)	1.11 (1.10, 1.11)
	1915–1919	1.35 (1.35, 1.36)	1.55 (1.54, 1.56)	1.15 (1.15, 1.16)
	1920–1924	1.48 (1.47, 1.49)	1.76 (1.75, 1.77)	1.20 (1.19, 1.21)
	1925–1929	1.62 (1.61, 1.63)	2.00 (1.99, 2.02)	1.26 (1.24, 1.27)
	1930–1934	1.78 (1.77, 1.79)	2.27 (2.25, 2.29)	1.32 (1.31, 1.33)
	1935–1939	1.97 (1.96, 1.98)	2.53 (2.51, 2.55)	1.39 (1.38, 1.40)
	1940–1944	2.13 (2.11, 2.14)	2.82 (2.80, 2.84)	1.45 (1.43, 1.46)
	1945–1949	2.30 (2.29, 2.32)	3.15 (3.13, 3.17)	1.51 (1.49, 1.52)
	1950–1954	2.51 (2.49, 2.52)	3.53 (3.50, 3.56)	1.57 (1.56, 1.59)

The reference groups for the age, period and cohort effects are the mean influences of all age, period and cohort groups combined, respectively. CI, confidence interval; RR, relative risk.

## Data Availability

All of the data are publicly available in this link: https://vizhub.healthdata.org/gbd-results/ (accessed on 5 October 2022).

## Supplementary Materials

The following supporting information can be downloaded at https://www.mdpi.com/article/10.3390/nu14235008/s1: Table S1: Temporal trends in nutritional deficiencies from 1990 to 2019 among the Chinese elderly as stratified by sex; Table S2: Autocorrelation evaluation in joinpoint regression models; Figure S1: Trends in age-standardized incidence rate per 100,000 person-years of (A) vitamin A deficiency, (B) iodine deficiency, and (C) protein-energy malnutrition with autocorrelation corrected among the elderly in China from 1990 to 2019 as stratified by sex.

## Abbreviations

AAPC, average annual percentage change; APC, annual percentage change; CI, confidence interval; RR, relative risk.
