# Supplementary material for: Temporal Trends in Incidence of Nutritional Deficiency among Older Adults in China: A Secondary Analysis of the Global Burden of Disease Study 1990–2019
Source: Nutrients. 2022 Nov 25;14(23):5008. doi: 10.3390/nu14235008 (PMC9738542; doi:10.3390/nu14235008)
Supplement: Supplementary file 1 [file nutrients-14-05008-s001.zip › nutrients-1993576-supplementary.pdf]

Table S1. Average percentage change with 95% CI of nutritional deficiencies, stratified by sex.

Both			Male			Female		
APC (95% CI)	P value	Period	APC (95% CI)	P value	Period	APC (95% CI)	P value	Period
Vitamin A deficiency								
-6.72 (-7.3, -6.13)	<0.001	1990-1998	-6.54 (-7.08, -6)	<0.001	1990-2000	-7.14 (-7.59, -6.69)	<0.001	1990-1999
-5.53 (-5.84, -5.22)	<0.001	1998-2013	-2.96 (-6.61, 0.83)	0.118	2000-2003	-5.04 (-5.34, -4.75)	<0.001	1999-2012
-2.82 (-6.37, 0.87)	0.125	2013-2016	-7.51 (-9.01, -5.97)	<0.001	2003-2009	-2.41 (-5.4, 0.67)	0.117	2012-2016
-8.02 (-11.08, -4.85)	<0.001	2016-2019	-5.54 (-6.03, -5.05)	<0.001	2009-2019	-7.22 (-9.95, -4.42)	<0.001	2016-2019
Iodine deficiency								
-0.07 (-0.13, -0.01)	0.027	1990-1996	0.18 (-0.09, 0.46)	0.178	1990-1995	-0.2 (-0.26, -0.13)	<0.001	1990-2000
-0.66 (-0.7, -0.62)	<0.001	1996-2004	-0.95 (-1.17, -0.74)	<0.001	1995-2000	-1.43 (-1.72, -1.13)	<0.001	2000-2004
-0.39 (-0.41, -0.37)	<0.001	2004-2017	-0.07 (-0.35, 0.21)	0.627	2000-2005	0.18 (0.12, 0.23)	<0.001	2004-2017
-1.5 (-1.68, -1.32)	<0.001	2017-2019	-1 (-1.06, -0.95)	<0.001	2005-2019	-1.94 (-2.41, -1.47)	<0.001	2017-2019
Protein-energy malnutrition								
2.17 (1.79, 2.54)	<0.001	1990-2000	3.65 (3.49, 3.81)	<0.001	1990-2000	0.24 (0.15, 0.33)	<0.001	1990-2006
0.4 (-0.72, 1.53)	0.469	2000-2005	0.71 (0.37, 1.06)	<0.001	2000-2006	4.77 (3.89, 5.66)	<0.001	2006-2010
3.91 (1.37, 6.51)	0.004	2005-2009	3.97 (3.28, 4.66)	<0.001	2006-2010	-2.86 (-3.26, -2.46)	<0.001	2010-2016
1.11 (0.74, 1.48)	<0.001	2009-2019	2.02 (1.87, 2.18)	<0.001	2010-2019	5.48 (4.68, 6.29)	<0.001	2016-2019

APC: annual percentage change; CI: confidence interval.

Table S2. Autocorrelation evaluation in joinpoint regression models.

	Both sexes combined		Male		Female	
	Durbin-Watson statistic	P value	Durbin-Watson statistic	P value	Durbin-Watson statistic	P value
Vitamin A deficiency	0.61	<0.001	0.83	<0.001	0.73	<0.001
Iodine deficiency	2.20	0.586	1.15	0.013	1.25	0.030
Protein-energy malnutrition	0.64	<0.001	0.89	<0.001	1.93	0.855

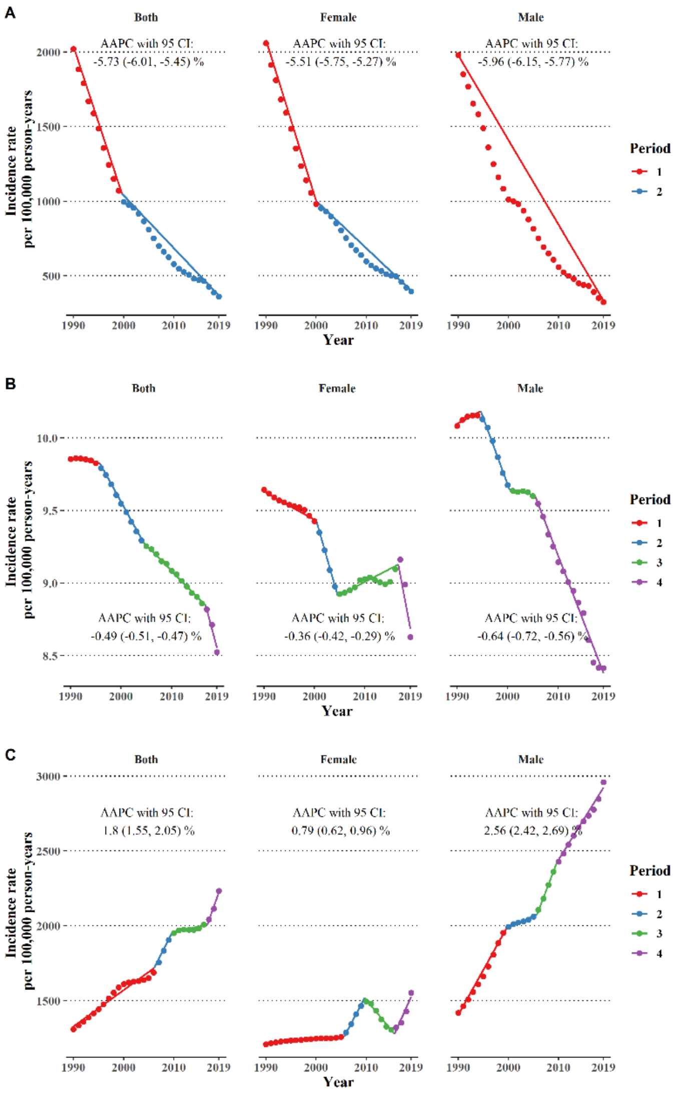

Figure S1. Trends in age-standardized incidence rate per 100,000 person-years of (A) vitamin A deficiency, (B) iodine deficiency, and (C) protein-

energy malnutrition with autocorrelation corrected among the elderly in China from 1990 to 2019, stratified by sex.
